# Clinical features of syndromic microphthalmia in two novel *RARB* variants

**DOI:** 10.1038/s41439-026-00345-3

**Published:** 2026-04-06

**Authors:** Yoshito Koyanagi, Hazuki Morikawa-Anzai, Tomoyo Yoshida, Makiko Tominaga, Yuichi Abe, Rika Kosaki, Keiko Matsubara, Maki Fukami, Sachiko Nishina

**Affiliations:** 1 Division of Ophthalmology, National Center for Child Health and Development, Tokyo, Japan; 2 Department of Ophthalmology, Graduate School of Medical Sciences, Kyushu University, Fukuoka, Japan; 3 Showa Medical University Northern Yokohama Hospital, Center for Clinical Genetics and Genomic Medicine, Kanagawa, Japan; 4 Showa Medical University Northern Yokohama Hospital, Children’s Center, Kanagawa, Japan; 5 Division of Neurology, National Center for Child Health and Development, Tokyo, Japan; 6 Center for Medical Genetics, National Center for Child Health and Development, Tokyo, Japan; 7 Department of Medical Genetics, National Center for Child Health and Development, Tokyo, Japan; 8 Department of Molecular Endocrinology, National Research Institute for Child Health and Development, Tokyo, Japan; 9 Division of Diversity Research, National Research Institute for Child Health and Development, Tokyo, Japan

**Keywords:** Genetic testing, Disease genetics

## Abstract

Here we describe unrelated Japanese patients with distinct novel heterozygous retinoic acid receptor beta (*RARB*) gene variants underlying syndromic microphthalmia-12: case 1 with a frameshift variant, c.1205_1206del, had bilateral microphthalmia, corneal opacity, anterior segment dysgenesis, widespread multiorgan anomalies, hypotonia and cognitive impairment; case 2 with a missense variant, c.844G>T had Peters anomaly, extreme microphthalmia, spasticity, profound psychomotor delay and refractory epilepsy. These findings highlight the need for *RARB* testing.

Microphthalmia, a heterogeneous group of disorders characterized by a reduced globe size, is often accompanied by other ocular malformations and extraocular anomalies. A nationwide survey of microphthalmia in Japan revealed that systemic diseases were present in 31% of all cases^1^. Syndromic microphthalmia-12 (MCOPS12; OMIM #615524) is a disorder primarily characterized by ocular malformations and various systemic and neurodevelopmental abnormalities, and it is caused by pathogenic variants in the retinoic acid (RA) receptor beta (*RARB*) gene. The RA pathway is crucial for organ development, including the brain and eyes, in humans and animals^2^. In target cells, RA binds to a heterodimer of the RA receptor (RAR) and retinoid X receptor (RXR); three RAR subtypes exist: RARA, RARB and RARG. These RAR–RXR dimers regulate target gene transcription by binding to RA response elements, a process facilitated by co-regulators and the RA-induced repositioning of RAR’s helix-12^3^. *RARB* encodes RAR beta, a ligand-activated nuclear receptor protein critical for orchestrating cell differentiation, proliferation and pattern formation during embryonic development. Variants in the *RARB* gene lead to a severe and progressive form of early-onset movement disorders, particularly dystonia. Intriguingly, recent genome-wide association studies have identified *RARB* as a candidate gene for strabismus, suggesting that genes linked to strabismus also may play a role in globe morphology^4^. So far, only a limited number of *RARB* pathogenic variants have been reported, contributing to a diverse clinical spectrum of MCOPS12^5–9^. Here, we describe two unrelated Japanese patients with novel *RARB* variants, which expand our understanding of the clinical and genetic features of MCOPS12.

## Case 1

**Fig. 1:**
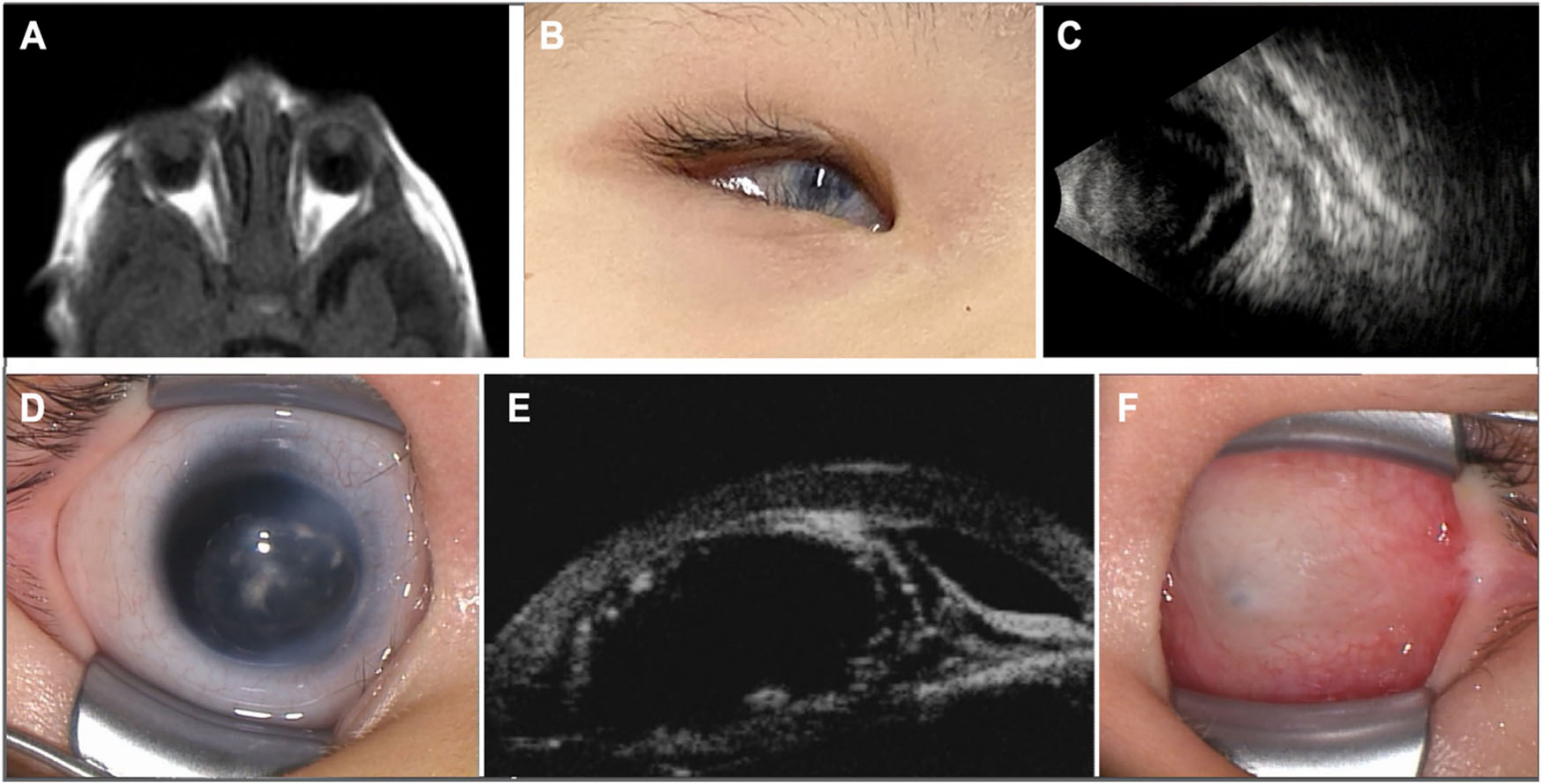
Ocular imaging findings of the two cases. **A** Magnetic resonance imaging of both eyes of case 1, acquired using a T1-weighted fluid-attenuated inversion recovery sequence. **B** Anterior segment photograph of the right eye in case 1. The patient exhibited bilateral microphthalmia, corneal opacity, anterior segment dysgenesis and left chorioretinal coloboma. The corneal diameters were approximately 5.0 mm in both eyes. **C** The left eye developed retinal detachment, which B-mode ultrasonography confirmed, suggesting a rhegmatogenous etiology potentially due to self-inflicted ocular pressure. **D** Anterior segment photograph of the right eye of case 2. **E** B-mode ultrasonography of the right eye of case 2. The initial ophthalmologic examination at 6 weeks of age revealed Peters anomaly type II in the right eye, characterized by central corneal opacity with iridocorneal and lenticulocorneal adhesions. The corneal diameter measured 9 × 9 mm in the right eye. **F** The left eye of case 2 exhibited extreme microphthalmia. The corneal diameter measured 1.5 × 1.5 mm in the left eye.

Case 1 is a 7-year-old Japanese girl, the second child of nonconsanguineous parents, who was born at term (38 weeks and 3 days of gestation) via vaginal delivery following frozen embryo transfer through in vitro fertilization. Her birthweight and head circumference were 2,870 g and 33.5 cm, respectively. The patient experienced vomiting since birth, which became bilious on day 3, leading to her transfer and admission to the Neonatal Intensive Care Unit. During this initial hospitalization, multiple congenital anomalies were identified, including bilateral microphthalmia, aniridia, corpus callosum hypoplasia, left diaphragmatic eventration (later confirmed as a diaphragmatic hernia postoperatively), duodenal obstruction with intestinal malrotation, and left renal agenesis. Throughout infancy, the patient required frequent hospitalizations for respiratory distress and feeding difficulties, often triggered by infections. By 5 years of age, the frequency of infection-related hospitalizations, including episodes of hypoglycemia, began to decrease, and her general systemic condition stabilized. Ophthalmic evaluation revealed bilateral microphthalmia, corneal opacity, anterior segment dysgenesis and a left chorioretinal coloboma (Fig. 1A, B). The corneal diameter was approximately 5.0 mm in both eyes. The right eye had light perception vision, and left pupillary closure was noted at the initial visit. Her left eye subsequently developed a retinal detachment, which was confirmed using B-mode ultrasonography, suggesting a rhegmatogenous etiology, potentially due to self-inflicted ocular pressure (Fig. 1C). Neurologically, she exhibited marked axial hypotonia, leading to unstable head control and the inability to sit independently. She had normal hearing but experienced insomnia, for which melatonin was prescribed. By 5 years of age, she acquired the ability to form three-word sentences. Of note, she was enrolled in Cure MCOPS12 (https://rarbmutation.org/), a registered nonprofit organization dedicated to improving the lives of children and families affected by MCOPS12. Through genetic diagnosis, parents can actively engage with international support groups and researchers, promoting a better understanding of the disease.

## Case 2

Case 2 was a 14-year-old Japanese boy, the second child of nonconsanguineous parents, who presented with severe ocular anomalies and neurodevelopmental delay. The initial ophthalmologic examination at 6 weeks of age revealed Peters anomaly type II in the right eye, characterized by central corneal opacity with iridocorneal and lenticulocorneal adhesions (Fig. 1D, E). The corneal diameter was 9 × 9 mm in the right eye and 1.5 × 1.5 mm in the left eye. The left eye exhibited extreme microphthalmia (Fig. 1F). The patient underwent a lensectomy and anterior vitrectomy of the right eye under general anesthesia using a 25-gauge system at 4.5 months of age. The entire lens and capsule were successfully removed, and the retina, optic disc and macula appeared grossly normal within the observable range. At 13 months of age, the patient underwent vitreous surgery in the left eye under general anesthesia using a 20-gauge system, with an infusion port placed at the 9 o’clock position. A superior corneal approach was used to remove the fibrovascular membranes and the anterior vitreous. The retinal folds caused by vitreoretinal traction resolved after dissection of the adherent strands, and the fundus became visible following membrane removal. The visual acuity could not be measured during the clinical course. Electroretinography (Neuropack, Nihon Kohden, performed according to the International Society for Clinical Electrophysiology of Vision protocol^10^) revealed detectable responses in the right eye, whereas the left eye showed nearly absent responses. Systemically, the patient exhibited profound psychomotor delay with the inability to achieve independent ambulation or expressive language. A neurologic examination revealed notable lower limb spasticity with multiple joint contractures, particularly affecting the knees and ankles. He also had refractory epilepsy with seizures, which remained poorly controlled despite multiple trials of different antiepileptic drugs. Behavioral disorders observed in the patient included autistic features, self-injurious behaviors and chronic insomnia. Unlike case 1, no major visceral malformations were identified through extensive systemic investigations.

To investigate the patients’ genetic backgrounds in both cases, genomic DNA was extracted from peripheral blood or saliva (from the father of case 1) using standard procedures. For case 1, single-proband whole-exome sequencing was performed using the SureSelect XT Human All Exon V6 platform. In case 2, panel sequencing of genes causing microphthalmia, including *ABCB6*, *ALDH1A3*, *BMP4*, *CRYBA4*, *GDF6*, *OTX2*, *RARB*, *RAX*, *SOX2*, *STRA6*, *TENM3* and *VSX2*, was performed at the Kazusa DNA Research Institute (Chiba, Japan) using short-read next-generation sequencing. Subsequent phenotype-driven filtering prioritized variants in *RARB* (NM_000965.5) as the leading candidate variants, identifying c.1205_1206del, p.(Leu402Hisfs*9) in case 1 and c.844G>T, p.(Gly282Cys) in case 2.

**Fig. 2:**
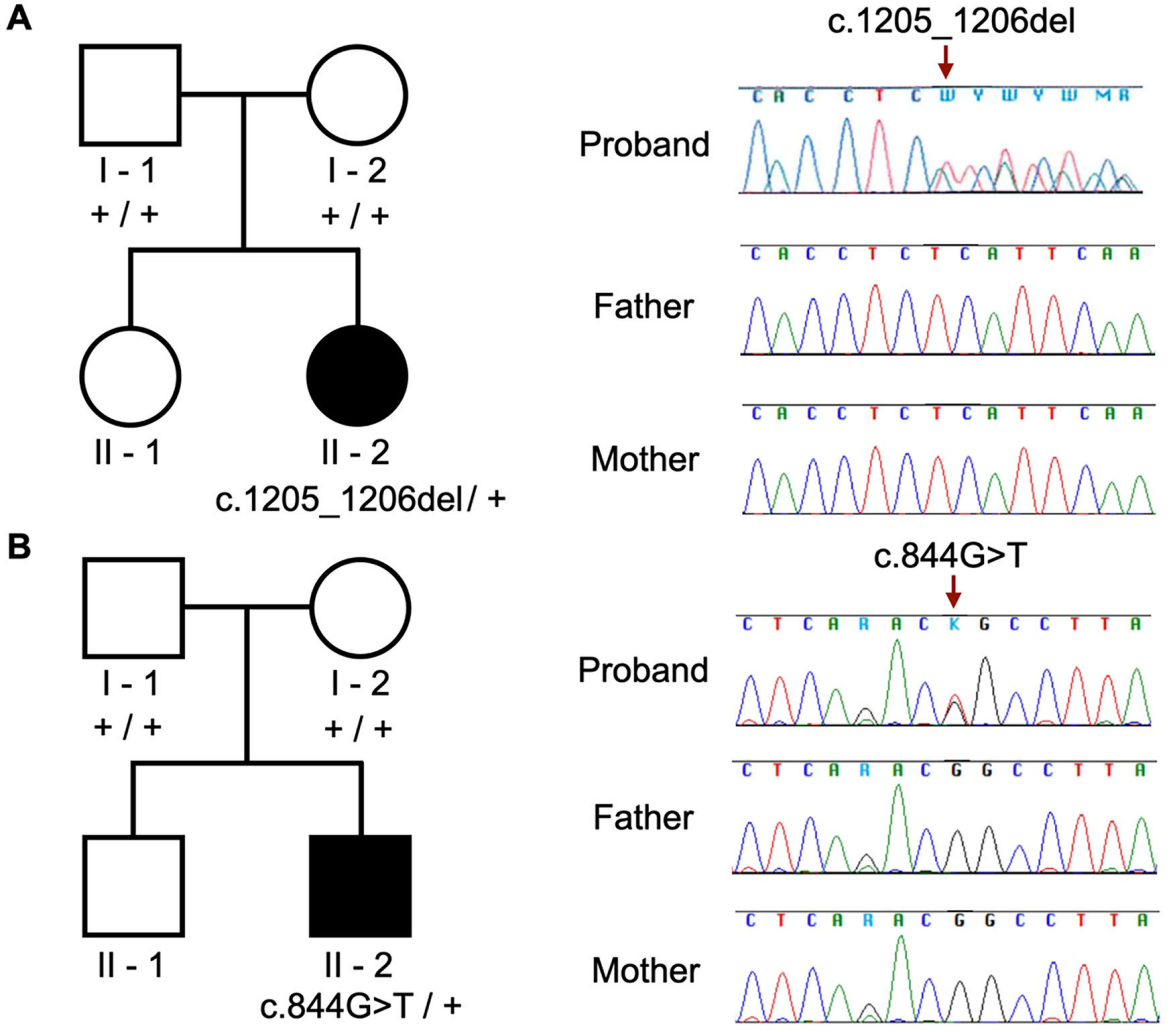
Novel *RARB* genetic variants in the patients. **A** Case 1 had a rare heterozygous frameshift variant in *RARB*, c.1205_1206del. No heterozygous variant was identified in the father or mother. **B** Case 2 had a rare heterozygous missense in *RARB*, c.844G>T. No heterozygous variant was identified in the father or mother.

We further validated the detected *RARB* variants using Sanger sequencing according to the standard protocol and co-segregation (Fig. 2). Specific primer sets were used for Sanger sequencing for targeted validation of the detected variants. The following primers were used for the *RARB* variant c.1205_1206del: forward primer 5′-TTTTCAACAAAAAGCCACCTGA-3′ and reverse primer 5′-CCTGGAACTGAAGGTACTGGG-3′. Conversely, the following primers were used for the *RARB* variant c.844G>T: forward primer 5′-GTGAAAGGGGGACAGACTGC-3′ and reverse primer 5′-CCATGTAAAACAACTCCAAAAGGGA-3′. Based on the Sanger sequencing data of the probands and parents, we identified two previously unreported heterozygous variants, c.1205_1206del and c.844G>T in the *RARB* gene, both of which were de novo (Fig. 2). For case 1, we provide additional visualization of the 2-bp deletion using Integrative Genomics Viewer snapshots (Supplementary Fig. 1).

The detected variants were interpreted according to the American College of Medical Genetics and Genomics guidelines^11^ and analyzed as described previously^12^. Common variants were filtered using the Genome Aggregation Database (https://gnomad.broadinstitute.org/)^13^ and Tohoku Medical Megabank Organization (4.7KJPN, https://jmorp.megabank.tohoku.ac.jp/)^14^. We cross-referenced variants against ClinVar, HGMD. Although c.1205_1206del is in ClinVar, it lacks peer-reviewed clinical documentation. The c.844G>T variant is absent from ClinVar and HGMD. Given its de novo occurrence at a residue where another substitution, such as c.844G>A, is established as pathogenic in an autosomal dominant disorder^7^, this variant is classified as autosomal dominant. Based on these findings, the identified variants were classified as pathogenic, confirming the genetic diagnosis of MCOPS12. Both were categorized as novel due to the absence of prior clinical descriptions in the medical literature.

Although their systemic and neurologic profiles varied, both patients exhibited severe anterior segment dysgenesis with microphthalmia and global motor delay. The former frameshift variant, c.1205_1206del, manifests as widespread multiorgan anomalies with hypotonia and cognitive impairment, whereas the latter missense variant, c.844G>T, is associated with central nervous system dysfunction, including refractory epilepsy, spasticity and absence of visceral anomalies. A review of 14 previously reported cases revealed universal bilateral microphthalmia (100%), global motor delay (100%) and intellectual disability (100%), whereas diaphragmatic hernias and congenital heart disease were observed in 57% and 43% of patients, respectively^5,6,9^. Chiari type 1 malformations and spasticity were reported in 55% and 70% of the cases, respectively. The phenotypic differences between the frameshift and missense variants probably reflect their distinct molecular effects. Frameshift variants often cause premature truncation of the protein, leading to loss of function and widespread multisystem involvement, as seen with visceral anomalies and cognitive impairment. By contrast, missense variants may preserve the protein’s overall structure but disrupt specific functional domains, resulting in more selective effects, such as central nervous system dysfunction without visceral anomalies. This variant-dependent pattern is supported by previous reports showing shared major features (for example, microphthalmia and motor delay) but differing frequencies of systemic versus neurologic findings^5–9^, suggesting the effect of variant types on clinical presentation. Both patients are still being followed, which may yield further findings as the disease progresses.

In conclusion, our findings expand the clinical spectrum of MCOPS12 and highlight the importance of *RARB* analysis in patients with complex ocular malformations accompanied by systemic and neurodevelopmental abnormalities.

## HGV database

The relevant data from this Data Report are hosted at the Human Genome Variation Database via Figshare at 10.6084/m9.figshare.hgv.3640 and 10.6084/m9.figshare.hgv.3643.

## Supplementary information

Integrative Genomics Viewer snapshot of the *RARB* variant in case 1.
