# Supplementary material for: Clinical features of syndromic microphthalmia in two novel RARB variants
Source: Hum Genome Var. 2026 Apr 6;13:11. doi: 10.1038/s41439-026-00345-3 (PMC13184296; doi:10.1038/s41439-026-00345-3)

Fig. S1. Integrative Genomics Viewer snapshot of the *RARB* variant in Case 1. This visualization confirms the heterozygous 2-bp deletion (c.1205_1206del) at the read level. Although the manuscript nomenclature follows GRCh38, this snapshot is based on the GRCh37 reference assembly used in the whole-exome sequencing pipeline for Case 1.

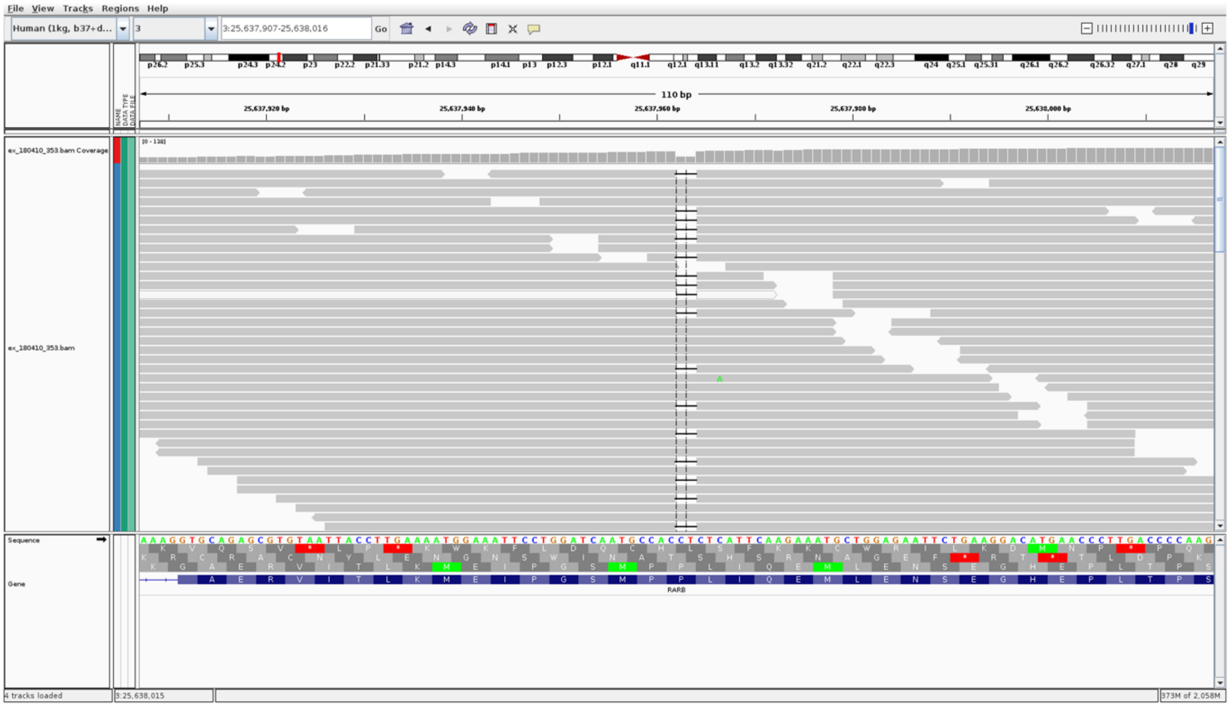

Supplement: Supplementary file 1 — Integrative Genomics Viewer snapshot of the RARB variant in case 1. [file 41439_2026_345_MOESM1_ESM.pdf]
